# Using a material database and data fusion method to accelerate the process model development of high shear wet granulation

**DOI:** 10.1038/s41598-021-96097-x

**Published:** 2021-08-13

**Authors:** Zheng Wang, Junjie Cao, Wanting Li, Yawen Wang, Gan Luo, Yanjiang Qiao, Yanling Zhang, Bing Xu

**Affiliations:** 1 Department of Chinese Medicine Informatics, School of Chinese Materia Medica, Beijing University of Chinese Medicine, No.11, North Third Ring East Road, Beijing, 100029 People’s Republic of China; 2 Beijing Key Laboratory of Chinese Medicine Manufacturing Process Control and Quality Evaluation, Beijing, 100029 People’s Republic of China

**Keywords:** Chemical engineering, Chemistry, Chemical engineering

## Abstract

High shear wet granulation (HSWG) has been wildly used in manufacturing of oral solid dosage (OSD) forms, and process modeling is vital to understanding and controlling this complex process. In this paper, data fusion and multivariate modeling technique were applied to develop a formulation-process-quality model for HSWG process. The HSWG experimental data from both literature and the authors’ laboratory were fused into a single and formatted representation. A material database and material matching method were used to compensate the incomplete physical characterization of literature formulation materials, and dimensionless parameters were utilized to reconstruct process variables at different granulator scales. The exploratory study on input materials properties by principal component analysis (PCA) revealed that the formulation data collected from different articles generated a formulation library which was full of diversity. In prediction of the median granule size, the partial least squares (PLS) regression models derived from literature data only and a combination of literature data and laboratory data were compared. The results demonstrated that incorporating a small number of laboratory data into the multivariate calibration model could help significantly reduce the prediction error, especially at low level of liquid to solid ratio. The proposed data fusion methodology was beneficial to scientific development of HSWG formulation and process, with potential advantages of saving both experimental time and cost.

## Introduction

In recent decades, wet granulation (WG) has been wildly used as a crucial process for manufacturing of oral solid dosage (OSD) forms. A survey by Leane et al.^1^ showed that WG was the most popular process choice compared with direct compression (DC), dry granulation (DG) and other technologies during the pharmaceutical development of over 80% of early-stage compounds. From manufacturing point of view, high shear wet granulation (HSWG), not only improves raw materials’ flowability and content uniformity, but also reduces dustiness and minimizes segregation, by which particles get ideal properties that adjust to subsequent processing such as compaction or coating^2^. It is the fact that desired granule attributes can be obtained by optimizing both the formulation and process conditions. To achieve this goal, lots of effort has been done on understanding multi-dimensional relationships among formulation properties, process parameters, and granule properties^2^. Nevertheless, wet granulation in practice has partly remained “more of an art than a science”^2,3^, due to the lack of knowledge in the multiple mechanisms^4^.

High shear wet granulation (HSWG) is an intricate particle size enlargement technique, which could be typically separated into three simultaneous rate processes, namely wetting and nucleation, consolidation and coalescence, and attrition and breakage^5^. In order to understand the complex mechanisms of the HSWG process, the nucleation regime map and the growth regime map were derived^3,4,6–8^. Some dimensionless parameters such as the drop penetration time and the spray flux, summarizing the key features of multiple process parameters, were brought forward^5,6^. Besides, population balance model (PBM) was developed to describe the evolution of granule properties over time, from single dimensional to multidimensional^9^. The complexity of HSWG further embraced advanced computational approaches such as discrete element model (DEM), computational fluid dynamics (CFD) and hybrid models, seeking for more accurate prediction of granulation behaviors^9–12^.

Although there was a large amount of effort focusing on modeling the HSWG process from first principles as described above, a rational approach for modeling or optimizing the wet granulation process was often realized by quality by design (QbD) experiments and statistical analysis in practice^2^. Until now, QbD principles have been applied to a considerable number of researches in HSWG process development^13–19^. For instance, Lee et al.^20^ optimized a high shear granulation process of a bilayer tablet based on QbD approaches, in order to achieve better physical stability via economical and simpler manufacturing processes. In another case, Fayed et al.^21^ investigated the critical influence of water amount and wet mixing time on both granules produced by high shear granulation and final tablets using the toolkit of QbD. From each QbD study, it was observed that the designed experiments often generated a solution to a particular product formulation at given granulation conditions. However, from various literature, information about the HSWG process can be acquired in multiple product formulations or experiments, at different granulation scales and conditions. An aggregation of available literature data through data fusion provides a possibility of obtaining more complete knowledge of the HSWG process.

Data fusion is a technique that integrates data and knowledge from multiple sources to produce a single model. There are three levels of data fusion, namely low-level (data level), mid-level (feature level) and high-level (decision level)^22^. So far, data fusion has been extensively applied in multisensor or multimodality environments^23,24^. The research of data fusion in pharmaceutical process development is at the preliminary stage. A data fusion model combing near infrared (NIR) features and process parameters for prediction of drug dissolution from controlled release multiparticulate beads was developed by Ibrahim et al.^25^. Casian et al.^26^ first applied a data fusion strategy to increase the quantitative ability of a process analytical technology (PAT) platform consisting of four sensors. Han et al.^27^ extracted 145 direct compressed oral disintegrating tablets (ODT) formulation data from 1218 articles in Web of Science database, and built a deep neural network (DNN) model to predict the disintegrating time of ODT formulations. To the best of our knowledge, there has been no study using the data fusion method to develop a HSWG process model.

In this work, we aim to establish a novel comprehensive dataset of high shear wet granulation from historical articles and to derive a statistical model for predicting the critical quality attribute (CQA) of granules. The diversity across the data sets from different HSWG related literature laid the foundation of data fusion, but it also brought forward challenges in transforming multiple data sets into unified representations. With regard to data alignment, the current paper provides the following contributions. From the aspect of formulation, the formulation materials and their mass fractions were recorded, but the material characterization would be inadequate or even missing. With the help of an established material database, several material matching rules were self-developed to give each formulation material a properties vector. Then, using the ideal mixing rule, the formulation properties were estimated. From the aspect of HSWG process, the process variables were described in many scales from the lab to the commercial. The dimensionless parameters regardless of the granulator scale were used to tackle this problem. To be specific, the Froude number (*Fr*) was used to mitigate variations of batch scales, and the theoretical maximum pore saturation ($S_{\max }^{^{\prime}}$) was used to explain the influence of binder addition on granule size^28^. Consequently, by using the formatted dataset with adequate information, a multivariate calibration model is more likely derived. In addition, the literature data sets may not have the same level of information quality or reliability. In order to enhance the prediction accuracy of HSWG process model, a few laboratory experiments designed by the Monte Carlo simulation and the random sampling were carried out. Two types of data, i.e. laboratory measurements and literature observations, were further integrated to build a more robust statistical model.

The rest of the paper was organized as follows. In “Methods” section, we explain the methodology of this research in detail. In “Results and discussion” section, the results of exploratory analysis, model development and validation were depicted and discussed. Finally, the conclusion and future research directions were given.

## Methods

### Literature data collection

The multivariate relationships of the HSWG process were assumed to be preserved in the data from the designed experiments of literature. To ensure the reliability, data were extracted from historical articles searched in the Web of Science and China National Knowledge Infrastructure database. Using “high shear wet granulation” as key words, the articles that employed water as binder agent of high shear wet granulation were collected. The criterions of selecting articles are described as follows. First, the articles should give the mass fraction and properties (if mentioned) of each formulation material. Some articles employed single material as the research object, and the corresponding mass fraction was regarded as 100 percent. Then, at least three process parameters such as the liquid to solid ratio, the impeller speed and the granulator scale were provided. Next, the median granule size *G*_50_ as the critical quality attribute must be measured and given, since the granule size had a significant impact on the subsequent processes (e.g. tableting) as well as the final product quality^29^. As a result, 10 articles were found to fit for the above requirements, and 143 pieces of experimental data were successfully extracted. These data have been fully documented in Table S1 waiting for further processing.

### Literature data alignment

#### Formulation properties estimation

**Figure 1:**
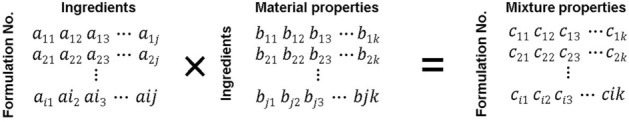
Prediction of formulation properties based on the ideal mixing rule.

Before the HSWG process, the ingredients of the formulation must be mixed uniformly. The mixture properties can be estimated using the ideal mixing rule^30^, as shown in Fig. 1. Linear combinations of physical properties of formulation materials were realized by multiplying the material properties matrix (*j* × *k*) by the formulation matrix (*i* × *j*) using MATLAB R2016b software (The MathWorks Inc., United States). In the collected 143 pieces of experimental data, 22 formulation materials including 19 pharmaceutical excipients and 3 APIs are involved in 17 formulations, as shown in Table S2. Hence, the formulation matrix was organized to contain 22 columns and 17 rows.

In the collected articles, there was a lack of characterization of input materials. The material properties matrix was built by taking advantage of an open access material database named iTCM^31^, in which 77 commonly used pharmaceutical excipients were respectively described by 18 physical properties. The mentioned material properties included bulk density (*D*_*a*_), tapped density (*D*_*c*_), true density (*D*_*t*_), powder porosity, interparticle porosity (*Ie*), Carr index (*IC*), cohesion index (*Icd*), Hausner ratio (*IH*), angle of repose (*a*), flowability time (*t*), moisture content (*HR*), hygroscopicity (*H*), proportion of particles smaller than 50 μm (*pf*), homogeneity index (*Iθ*), particle sizes *D*_10_, *D*_50_, *D*_90_, and particle size distribution width (*span*). The homogeneity index is the descriptor of uniformity of particle size distribution^32^. The hygroscopicity was determined by the increased percentage of the weight of the sample after placing the sample in the environment of constant temperature and humidity for 24 h^33^.

Considering the similarity between materials, the material properties matrix (22 × 18) was built by performing the following material matching operations. (1) If a formulation material had the same name and specifications with one pharmaceutical excipient included in the iTCM database, they were assumed to share the same physical properties; (2) in some articles, only the material name was given. The corresponding material properties were regarded as the weighted average of all excipients with the same name but different specifications in the database. (3) If a formulation material was outside the range of recorded materials in the iTCM database, its properties were supplemented with the most similar one in the database. For instance, the API of semi-fine acetaminophen whose particle sizes *D*_10_, *D*_50_ and *D*_90_ were 0.73, 2.75 and 5 µm, respectively^34^, was considered to bear the same properties with magnesium stearate that had very similar *D*_10_, *D*_50_ and *D*_90_ values (i.e. 1.21, 2.82 and 5.32 µm, respectively). The material matching methods applied for each formulation material are depicted in Table S2. 39 single materials from the iTCM database are involved during the material matching process, as shown in Table S3. The estimated material properties matrix can be seen in Table S4.

#### Process parameters processing

The process parameters from different articles were fairly not uniform. In order to make the process parameters comparable, they were processed from two aspects: agitation intensity and granules growth conditions.

##### Calculation of the Froude number

The granulator scales recorded in different articles were different, ranging from 0.25 to 600 L. In order to minimize the process variations caused by the scale, the Froude number (*Fr*) expressing the agitation intensity was calculated as follows^17,28^.

1 $$ Fr = \frac{{\omega^{2} (2R)}}{g}, $$

where *R* denotes the inner radius of granulator (m). *ω* is the impeller speed, i.e. the revolutions of impeller per second (rps), and *g* is the gravity constant. Considering the fact that the inner radius of granulator was not given in some articles, it was estimated by using a geometric formula based on an assumption of cylindrical granulator.

2 $$ R = \sqrt {\frac{V}{\pi H}} , $$

where *V* is the granulator scale (L) and *H* is the height of granulator. If the inner radius of granulator was not given, the equivalent *R* and *H* was utilized to calculate *R* from the granulator scale *V*. One qualified article^35^ as well as the granulator in our laboratory showed that *R* was equal to *H*.

In some articles, only the velocity of impeller blade tip was recorded. The impeller speed and the linear velocity of impeller could be transformed into each other with Eq. (3):

3 $$ v = 2\pi R\omega , $$

where *v* is the velocity of impeller blade tip (m/s).

##### Binder addition related parameters

In some literature, the binder addition process was described by total volume of water (mL) and total mass of formulation (g). While in other articles, the binder addition process was described by the binder addition rate (mL per minute) or the total addition time (minute). In this work, the liquid to solid ratio was calculated in order to make different forms of data comparable. Besides, the theoretical maximum pore saturation ($S_{\max }^{^{\prime}}$) was calculated to estimate the water saturation in dry powder bed without considering the consolidation of wet granules during the granulation process^6,36^.

4 $$ S_{\max }^{^{\prime}} = \frac{{w\rho_{s} \left( {1 - \varepsilon_{\min } } \right)}}{{\rho_{l} \varepsilon_{\min } }}, $$

5 $$ \varepsilon_{\min } = 1 - \frac{{\rho_{e} }}{{\rho_{p} }}, $$

where *w* is the mass ratio of liquid to solid, *ρ*_*s*_ is the bulk density of the solid particles, and *ρ*_*l*_ is the liquid density. *ε*_min_ is the minimum porosity the formulation reaches for that particular set of operating conditions, and it is determined by Eq. (5). For ungranulated powders, *ρ*_*e*_ is the envelope density of particles and *ρ*_*p*_ is the true density. The true density of the formulation mixtures was estimated by the ideal mixing rule on the basis of every ingredient’s true density and mass ratio. In this paper, the envelope density of particles was unknown, and it was substituted by the tapped density of the formulation mixtures^37^. In this way, the minimum porosity was estimated by measuring the dry-tapped porosity of the formulation.

### Laboratory data collection

#### Materials

In the formulation matrix mentioned in “Formulation properties estimation” section, there involved 3 kinds of HPMC, 6 kinds of lactose and 5 kinds of MCC, which were top three frequently used materials. Therefore, two cellulose materials and four lactose materials were selected from the iTCM material library to generate the simulated and experimental formulations. Microcrystalline cellulose (MCC) PH101 (lot No. 1545) was purchased from Asahi Kasei Chemicals Co., Ltd. (Japan). Lactose monohydrate Pharmatose^®^ 110M (Batch No. 1009CON), Pharmatose^®^ 200M (Batch No. 10095MW), Anhydrous lactose 21AN (Batch No. 1007NX8) and HPMC E15LV (Batch No. D011F6KL01) were purchased from Shanghai Chineway Pharmaceutical Technology Co., Ltd. (Shanghai, China). Lactose granulac^®^ 200 (lot No. L1535) was purchased from Molkerei MEGGLE Wasserburg GmbH&Co. KG. (Germany). The deionized water was used as the granulation liquid.

#### Experimental design

The experimental design was realized through a combination of Monte Carlo simulation and random sampling technique. It was assumed that each simulation formulation contained one type of cellulose materials and one type of lactose, and eight combinational forms of formulation could be obtained. For every combination, the mass fractions of one material were simulated to be varied from 0 to 100% at 1% increments, and the mass fractions of the other material were varied from 100 to 0% accordingly. As a result, a total of 808 simulation formulations were produced.

Two manipulated process parameters, i.e. liquid to solid (L/S) ratio and impeller speed were designed to carry out the granulation process. The number of simulated process parameters should match the number of simulated formulations. 808 L/S ratios were simulated randomly in the range from 0.025 to 1.8 g/g, where the upper and lower limits of L/S ratio were figured out from qualified literature data in “Literature data collection” section. The process settings must be within the capacity of lab granulator. The maximum and minimum impeller speeds of the lab granulator used was 1200 s^−1^ and 350 s^−1^, respectively. Hence, 808 impeller speeds as integers were simulated randomly in this range.

The operational boundaries of each variable in the experimental design are shown in Table 1. After the simulations were performed, 7 granulation experiments for modeling and 7 validation experiments were randomly selected from the 808 simulated conditions. The lab granulator had two interchangeable granulation bowls, i.e. 1 L and 2 L, the radii of which were 0.070 m and 0.087 m respectively. The granulator scale was a discrete variable, and it was randomly allocated to the designed experiments.

**Table 1:** Factors and limits in the experimental design.

Operation boundary	Formulation variable	Manipulated process variable		
	Mass fraction of cellulose material (%)	Liquid to solid ratio (g/g)	Impeller speed (s^−1^)	Granulator scale (L)
Upper limit	100	1.8	1200	2
Lower limit	0	0.025	350	1
Type	Continuous	Continuous	Continuous	Discrete

#### Granulation process

Granulation operations were performed using the high shear wet granulator (SHK-4, Xi’an Run Tian Pharmaceutical Machinery Co., Ltd., Xi’an, China). A three-blade impeller was located at bottom of the bowl and binder liquid was added by gently pouring from the top through a funnel. The total mass of formulation mixture was 150 g in the 1 L granulator, and 300 g in 2 L granulator. Dry mixing under 600 rpm of impeller speed with duration of 3 min was employed prior to wet granulation to ensure uniformity of material mixture. The wet mixing process lasted for 3 min, and the liquid addition and impeller speed were set according to the experimental design. The chopper speed was kept at constant at 1200 rpm, avoiding wet mass caking on the steel wall. The resultant granules were dried at 50 °C in an oven overnight. The granule size was determined using the sieve analysis. 6 different standard sieves, including 12, 20, 60, 80, 120 and 200 mesh, were employed. After vibrating for 1 min at 30 Hz using a vibration machine (ZNS-300, Beijing Xing Shi Lihe Technology Development Co., Ltd., Beijing, China), particles on each sieve were weighted. The granule size *G*_50_ was calculated by fitting the curve of mass cumulative size distribution.

### Multivariate process modeling

The partial least squares (PLS) was a prevalent data fusion algorithm^23,38^ and was employed to correlate the input and output data matrixes in this paper. PLS provides a tool by which considerable variations of initial data could be described by a few latent variables. The PLS regression model was built on the calibration set using SIMCA 13.0 software (Umetrics, Umea, Sweden). Coefficient of determination (*R*^2^), ability of prediction (*Q*^2^), accuracy and root mean square error of prediction (*RMSEP*) were used as model quality metrics. Formula of *R*^2^ and *Q*^2^ are as follows:

6 $$ R^{2} = 1 - SSE/SST, $$

where SSE is error sum of squares and SST is total sum of squares.

7 $$ Q^{2} = \left( {1.0 - \prod {\left( {PRESS/SS} \right)_{a} } } \right), $$

where *PRESS* is predicted residual error sum of square, *SS* is sum of squared deviations and subscript a represents a certain variable. Accuracy of PLS model is calculated by the following equation^27^:

8 $$ Accuracy = \frac{{Number(\left| {f^{\prime} - f} \right| \le Deviation)}}{All\;predictions}, $$

where *f’* is prediction value, *f* is real value, and *Deviation* is tolerance limit and the numerator gives number of acceptable deviations between the predicted value and the real value. *All predictions* are the total number of observations. *RMSEP* for the prediction set is denoted below.

9 $$ RMSEP = \sqrt {\sum {(Y_{obs} - Y_{pred} )^{2} /N} } , $$

where *Y*_*obs*_ and *Y*_*pred*_ refers to the predicted residuals for the observations in the prediction set. *N* is number of samples. *RMSEP* measures the predictive power of the model.

## Results and discussion

### Exploratory analysis on input material properties

**Figure 2:**
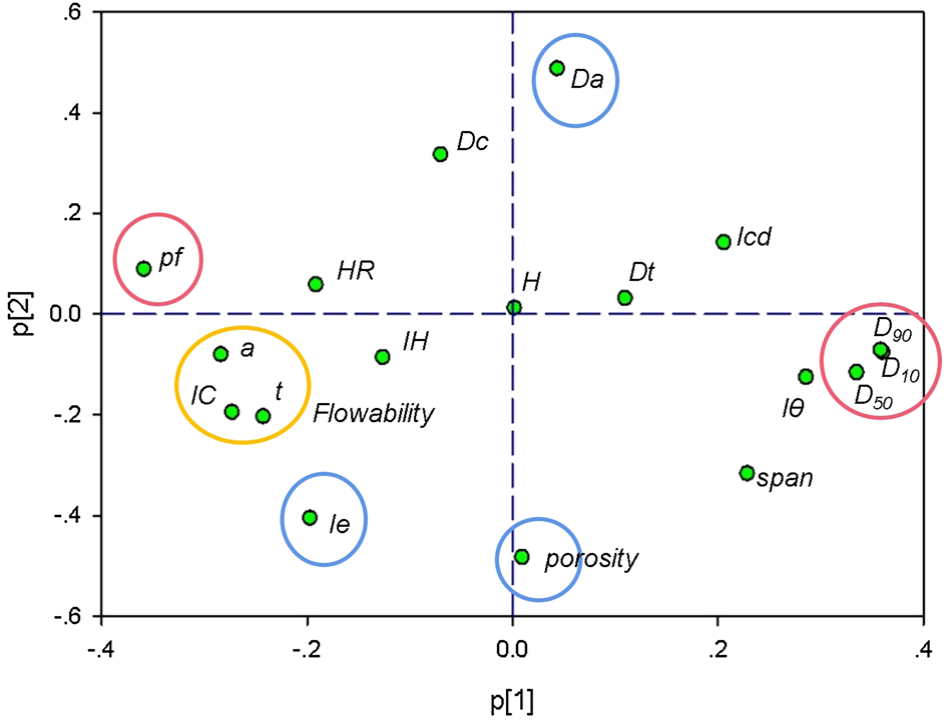
Loading plot of PC1 and PC2. The circles cluster related descriptors and describe the overarching physical property to which they contribute.

In order to find out if the collected formulations contain enough variations, the principal component analysis (PCA) was used to explore the material property space. PCA is an ideal technique to check the data structure in the latent space, and to judge the samples distribution characteristics. By using the ideal mixing rule, the formulation properties matrix described in “Formulation properties estimation” section was estimated. The formulation properties matrix (17 × 18) and the single material properties matrix (39 × 18) were combined as input material properties matrix (56 × 18) to be explored. Before PCA, variables were centered and scaled. The first two principal components were selected to summarize 56.1% of the total variation. Loadings illustrated the importance of descriptors towards principal components. As shown in Fig. 2, it can be seen that the particle sizes (*D*_10_, *D*_50_ and *D*_90_) and the homogeneity index (*Iθ*) are scattered in the positive side of x-axis. On the contrary, the proportion of particles smaller than 50 μm (*pf*) together with several flowability parameters such as Carr Index, flow time and angle of response in the yellow circle, are situated at the negative side of x-axis. These results proved that the principal component 1 (PC1) was predominated by particle size and powder flowability. While, the principal component 2 (PC2) was closely related with powder bulk density (*Da*) and porosity.

**Figure 3:**
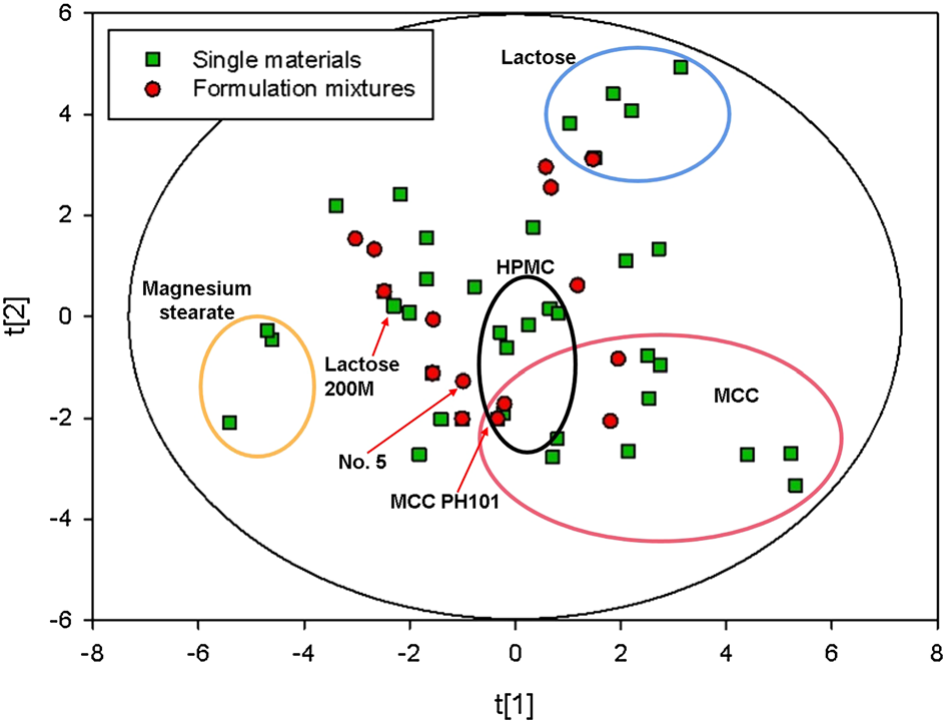
The PC1 vs PC2 score scatter plot. The color of the dots indicates different data sets where the green and red represent single materials and formulation mixtures respectively. The shape varies to discriminate where the data comes from: square means data extracted from iTCM database, circle represent data collected from literature respectively.

Figure 3 is the score plot, in which each dot represents a single material or a formulation mixture. As shown in this figure, there was no obvious cluster that could be recognized. All points stayed inside the 95% Hotelling T^2^ ellipse. MCC, HPMC, lactose and magnesium stearate were circled respectively. Approximately, the center of all MCC materials was located on the positive side of PC1-axis, suggesting that these kinds of powder tended to have relatively high particle size and low fine proportion. The magnesium stearate materials were on the negative fringe of PC1-axis, showing an opposite trend compared with MCC. Different kinds of lactose, being located on the positive side of PC2-axis, had high bulk density and low inter-particle porosity. The material characteristics of MCC and lactose could be confirmed in Naseem’s work^39^, in which MCC tended to have large average particle size while lactose had comparatively high bulk density. In Fig. 3, the red dots represent the formulation mixtures. The mixture properties of each formulation was a linear combination of the constituent single materials. For instance, No. 5 formulation in Table S1 was composed of 66.7% MCC PH101 and 33.3% lactose 200 M, and it was located in the straight line between the two pharmaceutical excipients.

### Process model derived from literature data

The critical quality attribute of the HSWG process (i.e. granule size *G*_50_) was at first tried to be predicted by a PLS model established on literature data. The input variables of the intended PLS regression model included 18 formulation material properties and 3 process parameters. For a given formulation, its mixture properties would be repeatedly arranged with related process parameters. Before model building, the outliers were detected to reduce the model prediction error. All 143 pieces of data were at first used to build an initial PLS model correlating the 21 independent variables and the CQA under 2 latent variables. 9 samples were found to be scattered outside the of Hotelling T^2^ ellipse at 95% confidence. These abnormal samples all used 100% (*w*/*w*) HPMC as the formulation material, and the liquid to mass ratios were approximate to 2. Besides, the resultant granules were over 6000 μm which were not common for pharmaceutical applications. The HPMC is a hydrogel forming polymer. When being reached by the water, the HPMC powders start to agglomerate due to hydrophobic interactions between the substituents on the polymeric chains, forming large aggregates^40^. At higher HPMC concentrations, such particular granulation mechanism may bring a big difficulty to provide a precise prediction of the granule size. By removing the outlier formulation No. 1, the rest of 16 formulations were randomly separated into 15 calibration formulations and 1 internal validation formulation (No. 17). The sample size of the calibration set and the internal validation set were 119 and 15, respectively. All variables were centered and scaled before process modeling.

The basic principle of choosing latent variables (LVs) is that when adding an extra LV into the PLS model, no obvious increase of both determinant coefficient (*R*^2^) and prediction ability (*Q*^2^) occur. As a result, 4 latent variables were capable to explain 87.1% of the total variation. The resultant *R*^2^ and *Q*^2^ were 0.743 and 0.718, respectively. *Accuracies* at different deviations directly showed the prediction performance. The majority (85.0% to be specific) of predictions had predictive errors below 300 μm, and only 36.2% of all predictions had predictive errors within 100 μm. Besides, the RMSEP was 152.6. These results indicated that the process model directly developed from literature data (Model 1) possessed low analysis efficiency. The experiment conditions in different articles varied a lot, which might lead to a model that was not robust enough to provide a precise prediction performance.

### Process model derived from integrated data

**Figure 4:**
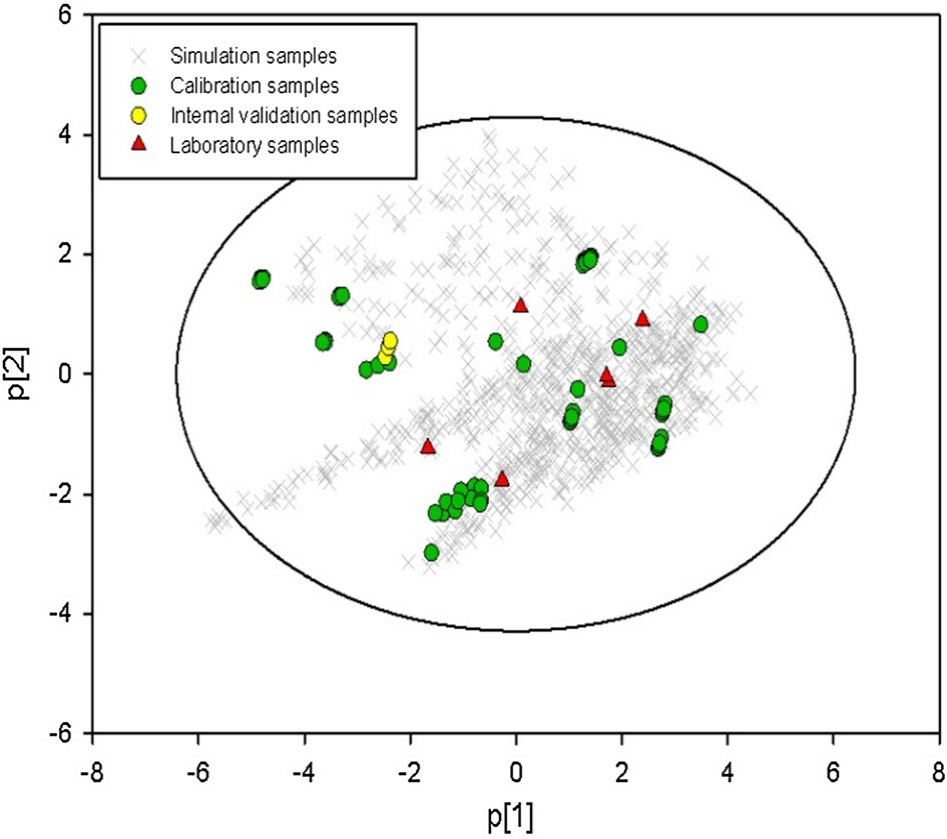
The LV1 vs LV 2 score scatter plot of PLS model 1.

In order to improve the model prediction performance for practical use, a few laboratory data were generated to supplement the literature data. According to the experimental design of “Formulation properties estimation” section and the formulation properties estimation methods in “Formulation properties estimation” section, the mixture properties of each simulation formulation were calculated by the ideal mixing rule, which gave a simulation formulation properties matrix **SX** (808 × 18). The Froude number was calculated by Eq. (1) on the basis of the radius of granulator and the impeller speed described in “Formulation properties estimation” section. 808 values of the Froude number were found in the range from 0.6 to 7.75. Due to the limitation of granulator in our laboratory, the maximum impeller speed of 1200 s^−1^ and the larger granulator radius of 0.087 m resulted the maximum *Fr* of 7.75, which was lower than the maximum value of *Fr* (i.e. 16.06) in collected literature. The corresponding $S_{\max }^{^{\prime}}$ parameter was estimated by Eq. (4) on the basis of the simulated parameters. 808 values of the $S_{\max }^{^{\prime}}$ were spread from 2.2 to 410.0. After that, a process parameters matrix **SP** (808 × 3) was built. The matrix **SX** and the matrix **SP** were combined to generate the matrix** S** (808 × 21). The samples of matrix **S** could then be projected into the latent variables space of PLS Model 1. Figure 4 shows the latent variables space by the first two LVs. The green circles represented calibration samples, and the yellow circles represented the internal validation samples. The grey cross were 808 simulated samples and the red triangles were 6 samples selected for laboratory granulation experiments. It can be seen that laboratory samples are distributed well among the calibration samples. Laboratory samples No. 2 and No. 6 appeared to be nearly overlapped in PLS scores space. The two samples had the same liquid to solid ratio and consisted of MCC and Lactose with the same mass fractions (i.e. 81% and 19%, respectively, *w*/*w*). The different types of Lactose (i.e. Lactose 200 M and Granulac 200) and *Fr* (i.e. 5.239 and 3.239, respectively) seemed to contribute less variation information to discriminate No. 2 and No. 6.

The laboratory granulation experiments were executed according to “Multivariate process modeling” section. The operational conditions and experimental results are illustrated in Table 2. The material mass fractions, the liquid to solid ratio and the impeller speed showed wide range. The resultant granule sizes had large distributions from 142 to 2017 μm. The sample No.7 failed to produce granule but got moist slurry, since the binder liquid was excessive. By adding six valid pieces of experimental data into the calibration set of PLS Model 1, an integrated and augmented calibration set containing 125 samples was established. The PLS Model 2 was built on this integrated data set. After the optimization of the number of LVs, 4 latent variables were capable to explain 86.5% of the total variation. The resultant *R*^2^ and *Q*^2^ were 0.710 and 0.680, respectively. *Accuracies* at deviations of 300 μm and 100 μm were 88% and 43%, respectively, which were higher than that of PLS Model 1. The *RMSEP* value decreased from 152.6 to 115.7, indicating the mean prediction error of granule size *D*_50_ was reduced greatly. Although the environment and operating conditions of our experiments were different from that of literature, the HSWG process was considered to follow the same process mechanism. By integration of the literature data and the laboratory data, the underlying features could be extracted through multivariate modeling technique. With the help of a small number of laboratory experiments, the prediction performance of process model could be improved efficiently.

**Table 2:** The results of laboratory granulation experiments for model improvement.

No.	Scale (L)	Component 1	Mass fraction (%)	Component 2	Mass fraction (%)	Liquid to solid ratio (L/S)	Froude number	Impeller speed (rpm)	*S*_max_	*G*_50_ (μm)
1	1	MCC PH101	64	Lactose 110M	36	0.858	4.212	1030	75.5	1400
2	1	MCC PH101	81	Lactose 200M	19	0.999	5.239	1149	82.7	1394
3	1	MCC PH101	95	Lactose 110M	5	1.333	3.178	895	92.2	2017
4	1	MCC PH101	38	Lactose 200M	62	0.315	0.975	496	41.3	205
5	1	HPMC E15LV	62	Anhydrous lactose	38	0.158	2.352	770	19.1	142
6	2	MCC PH101	81	Granulac 200	19	0.999	3.239	810	82.4	1054
7	1	MCC PH101	13	Lactose 110M	87	1.209	1.557	626	154.2	–

**Figure 5:**
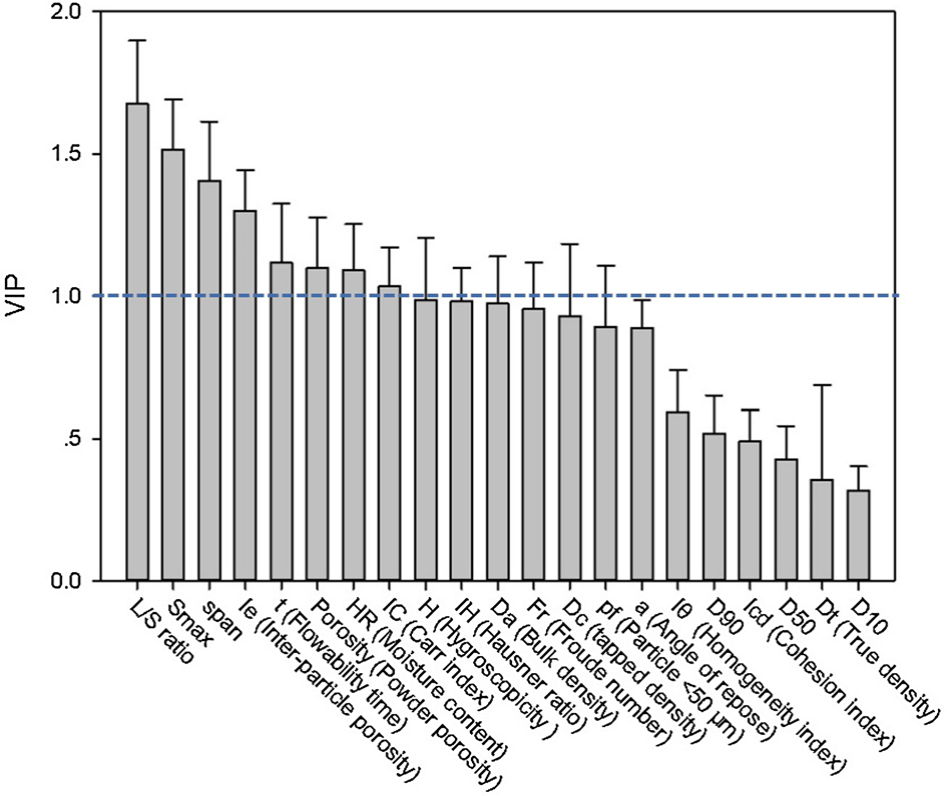
The variable importance in projection (VIP) plot of PLS Model 2.

**Figure 6:**
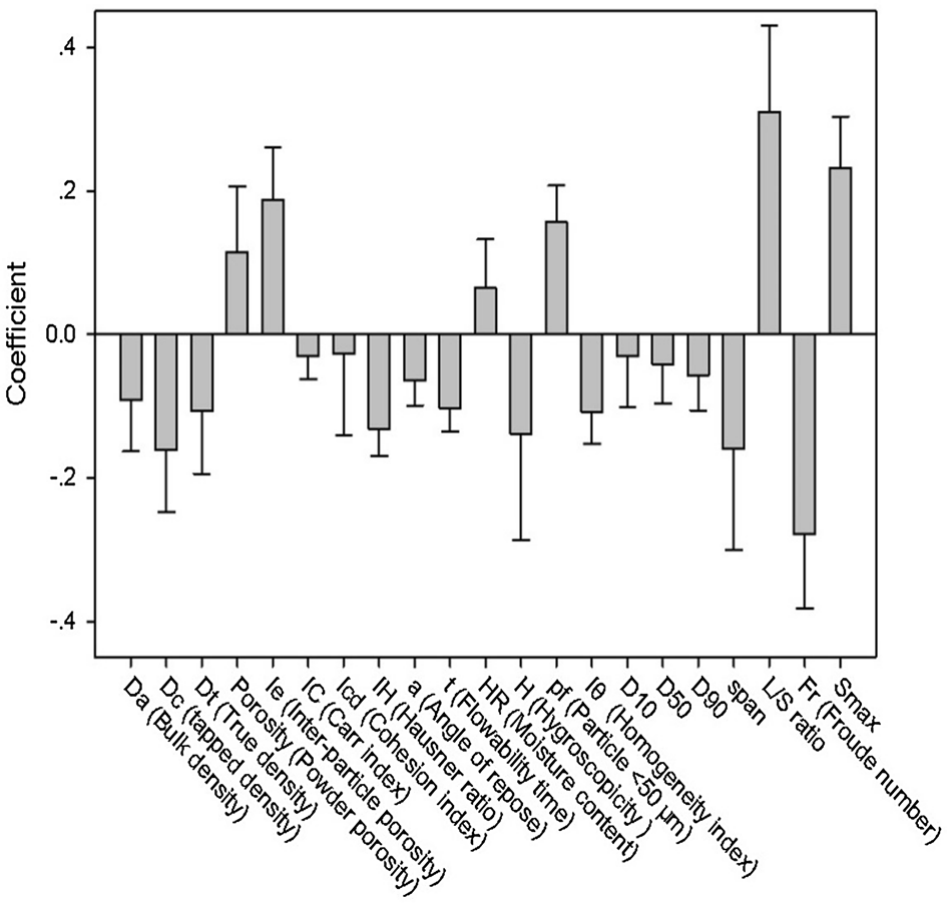
The coefficients plot of PLS Model 2.

The rationality of modeling results is further analyzed with the help of the variable importance in projection (VIP) plot and the coefficients plot, which are shown in Figs. 5 and 6, respectively. Variables with VIP values greater than 1 are considered to exert large impact on the model output. In Fig. 5, the first two important variables are L/S ratio and *S*, with VIP values equaling 1.68 and 1.52, respectively. The two variables also have large positive coefficient values, i.e. 0.31 and 0.23, respectively, as shown in Fig. 6. Parameter of L/S ratio revealed the amount of liquid binder added into the powder bed. $S_{\max }^{^{\prime}}$ is a measurement of liquid content. Similar to the maximum pore saturation, with the increase of $S_{\max }^{^{\prime}}$, the granulation experiences nucleation only to rapid growth or even over-wet mass. The rapid growth phase occurred under the conditions of high binder content, leading to larger granule size. Particularly, when $S_{\max }^{^{\prime}}$ exceeds the upper critical value of steady growth or induction growth process, slurry and mushy mass as the No. 7 experiment in Table 2, is likely to be obtained.

Another process parameter, the Froude number, was with large negative coefficient value (i.e. − 0.28). However, its VIP value was 0.79, which was smaller than 1. The Froude number was used to describe the impeller speed mitigating the error influenced by the granulator scale. In this work, the Froude number was dominated by the impeller speed, and had a negative impact on granule growth. High impeller speed would cause the attrition and breakage process and consequently resulted in the decrease of granule size. However, it should be pointed that the effect of impeller speed on granule size was a two-way regulation, a combination of granule consolidation and breakage dynamic equilibrium^28,41^. At the early stage of granulation, binder liquid inside the capillary of wet granule was squeezed out to the surface by shear force, promoting coalescence and consolidation process. With impeller speed increasing, shear force sharply rises, leading to intensive breakage of granules due to collisions in the granulator. As a result, the sophisticated mechanism of particle–particle and particle-binder interactions would be difficult to fit.

As shown in Fig. 5, VIP values of the majority of material properties are smaller than 1. This indicated that the input material properties had relatively less influence on the granule size, compared with process parameters. *span* was a vital material property with VIP value of 1.40, and it had a negative impact on granule size since its regression coefficient was − 0.16. This tendency was similar to Hounslow’s work^42^, in which a bimodal particle size distribution would favor coalescence through a layering mechanism of smaller particles onto the surface of larger ones, leading to an uneven growth process.

The porosity attributes of powder bed, such as powder porosity (VIP = 1.10) and inter-particle porosity (VIP = 1.16), were also the influential material properties for the granulation process. It has been reported that the penetration time of binder liquid into powder bed would be shorter with porous materials^43^, triggering the nucleation process and promoting the wetting interaction between raw materials and binder.

### Experimental validation of process model

According to “Experimental design” section, the external validation experiments were designed to verify the effectiveness of PLS Model 2. The experimental results are shown in Table 3. Similar to the No. 7 experiment in Table 2, the No. 14 experiment produced slurry and mushy mass and failed to obtain granules. This could be explained by the extraordinary $S_{\max }^{^{\prime}}$ value (i.e. 107.1) over than 100. The *RMSEP* values of external validation set calculated from PLS Model 1 and Model 2 were 284.3 and 149.2, respectively. The No. 12 experiment was modified by using 74.3% MCC PH101 and 25.7% HPMC E15LV as Component 1, producing a triple-component formulation to test the robustness of prediction. By using PLS Model 2, the absolute prediction error for No. 12 experiment was 36 μm, which was smaller than *RMSEP*. These results demonstrated that PLS Model 2 provided improved prediction ability.

**Table 3:** The results of external validation experiments.

No.	Scale (L)	Component 1	Mass fraction (%)	Component 2	Mass fraction (%)	Liquid to solid ratio (L/S)	Froude number	Impeller speed (rpm)	*S*_max_	Y_obs_ *G*_50_ (μm)	Y_pre_ (μm)
8	2	MCC PH101	64	Granulac 200	36	0.396	1.7	587	39.3	424	458
9	1	MCC PH101	81	Lactose 200M	19	0.862	2.878	852	71.4	1088	774
10	1	MCC PH101	95	Lactose 110M	5	0.882	2.619	812	61.0	978	798
11	1	HPMC E15LV	62	Anhydrous lactose	38	0.296	1.7	655	35.8	207	198
12	1	MCC PH101 (74.3%), HPMC E15LV(25.7%)	70	Anhydrous lactose	30	0.299	5.239	1149	30.6	194	158
13	2	MCC PH101	64	Granulac 200	36	0.658	2.212	670	65.2	641	632
14	1	MCC PH101	38	Lactose 200M	62	0.816	7.001	1200	107.1	–	–

Experiments No. 9, No. 10 and No. 13 employed relatively large liquid to solid ratios that were 0.862, 0.882 and 0.658, respectively, and the resulted granule sizes of *D*_50_ were 1088, 978 and 641 μm, respectively. The average absolute prediction error of the three experiments was 167.6 μm. By contrast, Experiments No. 8, No. 11 and No. 12 employed relatively small liquid to solid ratios that were 0.396, 0.296 and 0.299, respectively, and the resulted granule sizes of *G*_50_ were 424, 207 and 194 μm, respectively. The average absolute prediction error of the latter three experiments was 26.3 μm. These results confirmed the fact that larger L/S ratios was responsible for larger granule sizes. At low level of L/S ratio, the relatively small model prediction error would be acquired by PLS Model 2. In addition, the formulations in experiments No. 11 and No. 12 contained 62% and 25.7% of HPMC, respectively. The absolute prediction errors for experiments No. 11 and No. 12 were 9 μm and 36 μm, respectively, which were smaller than the average prediction error of the improved model. These results indicated that when the concentration of HPMC were not very high, favorite prediction performance could be obtained.

## Conclusion

In this paper, the partial least squares regression was used to build a formulation-process-quality model for the high shear wet granulation process. A material database of pharmaceutical excipients was used to estimate physical properties of HSWG formulation, and dimensionless parameters were utilized to reconstruct process variables at different granulator scales. The experimental data of HSWG process from two sources, i.e. literature and the authors’ laboratory, were fused into a single representation. Results demonstrated that incorporating a small number of laboratory data into the multivariate calibration model could help significantly reduce the prediction error. The proposed modelling approach proved three innovative ideas as follows.

1. Pharmaceutical materials belonging to the same category or possessing the same specifications had similar physical properties. The material database owned complete material properties data and could help maximize the material information during process model development.
2. The formulation data collected from different articles generated a formulation library, which was full of diversity and laid the foundation for process model generalizability.
3. The process model developed from literature data could be migrated to our laboratory conditions with the help of only a few laboratory experiments, the run number of which was less than that of traditional design of experiment. This led to savings in terms of both experimental time and cost.

However, a practical limitation to the multivariate calibration like PLS regression is that limited extrapolation is allowed beyond the scope of training data. In the future, more data pertaining to the HSWG process could be accumulated continuously, in order to strengthen the latent phenomena. Data with binders other than water may also be incorporated into the process model, and it is expected whether attractive new features will be discovered. The material database and data fusion methodology can be used in other scenarios, facilitating the scientific development of pharmaceutical formulation and process.

## Supplementary Information

Supplementary Information.

## Abbreviations

OSD: Oral solid dosage
DC: Direct compression
DG: Dry granulation
HSWG: High shear wet granulation
CQA: Critical quality attribute
DEM: Discrete element model
CFD: Computational fluid dynamic
QbD: Quality by design
CPP: Critical process parameter
API: Active pharmaceutical ingredient
ANN: Artificial neural network
TCM: Traditional Chinese medicine
PBM: Population balance model
ODT: Oral disintegrating tablet
DNN: Deep neural network
SSE: Error sum of square
SST: Total sum of square
SS: Sum of squared deviation
MVA: Multivariate analysis
PCA: Principle component analysis
PLS: Partial least squares
*Fr*: Froude number
$S_{\max }^{{\prime}}$: Theoretical maximum pore saturation
LV: Latent variable
MCC: Microcrystalline cellulose
HPMC: Hydroxypropyl methylcellulose
VIP: Variable importance in projection
RMSEP: Root mean square error of prediction
PC: Principle component
